# Antimicrobial Activities and Time-Kill Kinetics of Extracts of Selected Ghanaian Mushrooms

**DOI:** 10.1155/2017/4534350

**Published:** 2017-10-29

**Authors:** Theresa Appiah, Yaw Duah Boakye, Christian Agyare

**Affiliations:** Microbiology Section, Department of Pharmaceutics, Faculty of Pharmacy and Pharmaceutical Sciences, Kwame Nkrumah University of Science and Technology, Kumasi, Ghana

## Abstract

The rapid rise of antimicrobial resistance is a worldwide problem. This has necessitated the need to search for new antimicrobial agents. Mushrooms are rich sources of potential antimicrobial agents. This study investigated the antimicrobial properties of methanol extracts of* Trametes gibbosa*,* Trametes elegans*,* Schizophyllum commune*, and* Volvariella volvacea*. Agar well diffusion, broth microdilution, and time-kill kinetic assays were used to determine the antimicrobial activity of the extracts against selected test organisms. Preliminary mycochemical screening revealed the presence of tannins, flavonoids, triterpenoids, anthraquinones, and alkaloids in the extracts. Methanol extracts of* T. gibbosa, T. elegans, S. commune*, and* V. volvacea* showed mean zone of growth inhibition of 10.00 ± 0.0 to 21.50 ± 0.84, 10.00 ± 0.0 to 22.00 ± 1.10, 9.00 ± 0.63 to 21.83 ± 1.17, and 12.00 ± 0.0 to 21.17 ± 1.00 mm, respectively. The minimum inhibitory concentration of methanol extracts of* T. gibbosa, T. elegans, S. commune*, and* V. volvacea* ranged from 4.0 to 20, 6.0 to 30.0, 8.0 to 10.0, and 6.0 to 20.0 mg/mL, respectively. Time-kill kinetics studies showed that the extracts possess bacteriostatic action. Methanol extracts of* T. gibbosa, T. elegans, S. commune*, and* V. volvacea* exhibited antimicrobial activity and may contain bioactive compounds which may serve as potential antibacterial and antifungal agents.

## 1. Introduction

Infectious diseases pose serious threats to the existence, health, and survival of mankind [1]. The World Health Organization (WHO) survey in 2008 indicated that infectious diseases caused 32% of deaths worldwide with 68% of the deaths occurring in Africa [2]. Infectious diseases still account for a great proportion of death globally and in some regions remain the most important cause of ill health [2]. Apart from affecting the health of individuals directly, infectious diseases have heavy impact on whole societies and economies [3]. The discovery of penicillin and subsequent development and synthesis of other antibiotics had been a milestone in the history of medicine. However, this medical breakthrough is being lost to the development and rapid spread of bacterial resistance to antimicrobial agents [4].

Globally, the emergence of antimicrobial resistant bacterial strains [5] is increasingly limiting the potency of current drugs and significantly causing failure of treating infections [6]. This situation shows that the potencies of prevalent antibiotics are decreasing steadily [7]. Therefore, there is a great need to develop novel drugs to combat pathogenic microorganisms that have developed widespread microbial resistance to antibiotics [8]. Since multidrug resistance of microorganisms is a major medical concern, screening of natural products in search for new antimicrobial agents is the need of the hour [9]. The use of natural products has been extremely successful in the discovery of new medicine, and mushrooms could be a source of natural antimicrobials [10].

Reports indicate that mushrooms contain many biologically active components that offer health benefits and protection against diseases [11] and are responsible for their antitumor [12], anti-inflammatory [13], antioxidant [14], and antimicrobial activities [15]. Mushrooms have been reported in several studies to be one of the most promising sources for obtaining natural compounds that can act as anti-infective agents. Some mushrooms, including* Pleurotus ostreatus* [16, 17],* Ganoderma lucidum* [18],* Russula delica* [19],* Phellinus hartigii* [20], and* Stereum ostrea* [21], have been reported to exhibit wide range of antimicrobial activity against different Gram-positive, Gram-negative bacteria, and also fungi. However, the antimicrobial properties of some mushrooms have not been studied, while others have limited data as far as their antimicrobial activity is concerned. This has necessitated the need for the continuous screening of mushrooms for their antimicrobial activities.

*Trametes gibbosa* (Pers.) Fr.,* Trametes elegans* (Spreng. Fr.) Fr.,* Schizophyllum commune* Fr., and* Volvariella volvacea* are mushrooms belonging to the phylum Basidiomycota that are commonly found growing in Ghana.* T. elegans* is commonly known for its ability to degrade lignin [22]. However, its antibacterial and antioxidant activities have been reported [14, 23].* T. gibbosa* has been found to exhibit antibacterial [24], antioxidant [25], and cytotoxic activity [26]. Mbayo et al. [27] reported that methanol extract of* T. gibbosa* exhibited activity against* Pseudomonas aeruginosa*,* Staphylococcus aureus*,* Streptococcus pneumoniae*, and* Shigella sonnei*, with MIC values of 1.25, 2.5, 5, and 2.5 mg/mL, respectively.* S. commune* has long been acknowledged for its medical importance and have been reported to exhibit antioxidant [28], antitumor and immune-modulating [29], anti-inflammatory [30, 31], and antimicrobial [32] activities. Acetone and ethyl alcohol extracts of* S. commune* Fr. have been reported to exhibit activities against* Escherichia coli* ATCC 25922,* Staphylococcus aureus*, and* Pseudomonas aeruginosa* ATCC 27857 via the agar diffusion method [33].* V. volvacea* is an edible mushroom that has been reported to exhibit antioxidant [31, 34], hepatoprotective [35], anticancer [36, 37], immune-modulatory [38], and antimicrobial [39, 40] activities.* V. volvacea* is reported to exhibit activity against* Bacillus subtilis*,* Proteus vulgaris*, and* Candida albicans* [41]. Ayodele and Idoko [42] reported that pure culture of* V. volvacea* collected from the grass land area of Kogi State, Nigeria, showed activity against* E. coli* and* S. aureus*. However, studies on the antimicrobial properties of mushrooms in Ghana are very limited. This study therefore investigated four Ghanaians mushrooms including fruiting bodies of* Trametes gibbosa* (Pers.) Fr.,* Trametes elegans* (Spreng. Fr.) Fr.,* Schizophyllum commune* Fr., and* Volvariella volvacea* for their antimicrobial properties.

## 2. Materials and Methods

### 2.1. Collection of Mushroom Samples

Fruiting bodies of* T. gibbosa*,* T. elegans*,* S. commune*, and* V. volvacea* were collected from farms and forests in Ayeduase (latitude 6°40′33N, longitude 1°33′36W, and altitude 252 m) in the Ashanti Region, Ghana, from June to November 2012, from their natural habitats. These mushrooms were authenticated by Mr. A. K. Apetorgbor, a mycologist in the Department of Theoretical and Applied Biology, Kwame Nkrumah University of Science and Technology (KNUST), Kumasi, Ghana, and voucher specimens (KNUST/HMI/2014/S005, KNUST/HMI/2014/S006, KNUST/HMI/2014/S007, and KNUST/HMI/2014/S009, resp.) deposited in the Herbarium of Department of Pharmacognosy, KNUST, Kumasi, Ghana.

### 2.2. Preparation of Methanol Extracts

The fruit bodies of the mushrooms were dried in an oven (Gallenkamp, London, UK) at 30°C for two hours to a constant weight. The various mushroom samples were ground to fine powder using a lab mill machine (Christy and Norris, Chelmsford, UK). Two hundred grams of each sample was weighed and successively extracted with 1 L each of 70% v/v methanol. The mixture was allowed to stand at room temperature (28 to 30°C) for three days with frequent agitation and homogenized and the supernatant was filtered using Whatman filter paper (number 10) (Sigma-Aldrich, Michigan, USA). The filtrates were concentrated in a rotary evaporator (Rotavapor BÜCHI R-200 with heating bath B-490, Büchi, Konstanz, Germany) at 40°C under reduced pressure and lyophilized. The yield of the extract, related to the dried powdered mushroom materials of* T. gibbosa, T. elegans, S. commune*, and* V. volvacea*, was 28, 25, 20, and 13% w/w, respectively. The extracts were kept in air tight containers, labelled, and stored in a desiccator until required for use.

### 2.3. Mycochemical Screening

The methanol extracts of* T. gibbosa, T. elegans, S. commune*, and* V. volvacea* were screened to detect the presence or otherwise of secondary metabolites such as tannins, flavonoids, triterpenoids, and alkaloids, following standard procedures [43, 44].

### 2.4. Thin-Layer Chromatography (TLC) of Methanol Extracts

The methanol extracts of* V*.* volvacea*,* T*.* gibbosa*,* T*.* elegans*, and* S*.* commune* were investigated using TLC described by Marica et al. [45]. Three hundred milligrams (300 mg) of each extract was dissolved in 2 mL chloroform in a beaker and applied as spots with the aid of capillary tubes on a silica-gel coated plate (Qingdao Marine Chemical Plant, Qingdao, China) about 1 cm from the base. The spotted plates were run in a developed solvent system of 100% chloroform. The developed plate was observed under ultra-violet light using both short and long wavelengths (254 and 365 nm) and then sprayed with anisaldehyde (Sigma-Aldrich, London, UK) in order to reveal compounds present in the extracts. Distances between the spots were measured and the retention factor (*R*_*f*_) values were calculated, using the following:

(1) $$\begin{array}{c} R_{f}\text{ value}=\frac{\text{Distance moved by the compound}}{\text{Distance moved by the solvent front}}. \end{array}$$

### 2.5. Culture Media and Reference Antibiotics

Sabouraud dextrose agar, nutrient agar, and broth were purchased from Oxoid Limited, Basingstoke, UK. Ciprofloxacin (98% HPLC) and ketoconazole (98% HPLC) were procured from Sigma-Aldrich, Michigan, USA.

### 2.6. Test Organisms

Pure culture of* Escherichia coli* ATCC 25922,* Pseudomonas aeruginosa* ATCC 4853,* Klebsiella pneumoniae* ATCC 33495,* Salmonella typhi* ATCC 19430,* Streptococcus pyogenes* ATCC 19615* Staphylococcus aureus* ATCC 25923,* Enterococcus faecalis* ATCC 29212,* Bacillus subtilis* NTCC 4853, and* Candida albicans* ATCC 10231 were obtained from the Microbiology Section, Department of Pharmaceutics, Faculty of Pharmacy and Pharmaceutical Sciences, Kwame Nkrumah University of Science and Technology (KNUST), Kumasi, Ghana. Pure cultures of* Aspergillus niger*,* Aspergillus flavus*, and* Aspergillus tamarii* were obtained from Department of Animal and Crop Science, Faculty of Agriculture, Kwame Nkrumah University of Science and Technology (KNUST), Kumasi, Ghana.

### 2.7. Determination of Antimicrobial Activity Using the Agar Well Diffusion Method

The antimicrobial activity was evaluated using the agar well diffusion method described by Agyare et al. [46]. Twenty millilitres of sterile nutrient agar and potato dextrose agar stabilized at 45°C for 15 minutes were seeded with 100 *μ*L of 1 × 10^6^ colony forming units (CFU)/mL of test bacteria and fungus, respectively, and aseptically poured into a sterile Petri dish and allowed to set. Four wells (8 mm) equidistant from each other were created with a sterile cork borer (number 4). The wells were filled with 200 *µ*L of 7.5, 10, 20, and 30 mg/mL of methanol extracts of the mushrooms dissolved in sterile distilled water. The plate was made to stand on the bench for 30 minutes to 1 h to allow diffusion of the extract. The zones of growth inhibition (including diameter of well) were measured after 24 h of incubation at 37°C for bacteria and 72 h after incubation at 28°C for the fungus. Ciprofloxacin and ketoconazole (Sigma-Aldrich, Michigan, USA) were used as reference antimicrobial agents against test bacteria and fungus, respectively. The procedure was performed in independent triplicates and the mean zones of growth inhibition were determined.

### 2.8. Determination of Minimum Inhibitory Concentration (MIC)

The minimum inhibitory concentrations (MICs) of methanol extracts of* T. gibbosa, T. elegans, S. commune*, and* V. volvacea* against the test organisms were determined using the broth microdilution method described by Agyare et al. [46]. Each well of a 96-well microtiter plate was filled with 100 *µ*L double strength nutrient broth. Stock solutions of extracts within the range of 0.25 to 50 mg/mL were prepared and 20 *µ*L of inoculum (1 × 10^6^ CFU/mL) was added to each well. The plate was incubated at 37°C for 24 h. After incubation, 20 *µ*L of 3-(4,5-dimethylthiazol-2-yl)-2,5-diphenyltetrazolium bromide (MTT) (1.25 mg/mL) was added to each well. The plate was incubated again at 37°C and growth was observed as purple coloration, while clear/yellow coloration indicated no growth after 30 min of incubation. The MIC was determined as the lowest concentration which showed no visible growth upon the addition of MTT. Ketoconazole at concentration ranging from 1.0 to 10.0 *µ*g/mL and ciprofloxacin at concentration range of 0.01 to 1.0 *µ*g/mL were used as reference standards. The procedure was performed in independent triplicates to validate the results.

### 2.9. Determination of Minimum Bactericidal and Fungicidal Concentrations

The minimum bactericidal concentration (MBC) and minimum fungicidal concentration (MFC) of methanol extracts of* T. gibbosa, T. elegans, S. commune*, and* V. volvacea* against the test organisms were determined using the method described by Cos et al. [47]. Each well of a 96-well microtiter plate was filled with 100 *µ*L double strength nutrient broth. A specified volume of the stock solution was added to each well to obtain a twofold serial dilution of the extract within the range of 1 to 50 mg/mL. A volume of 20 *µ*L of 1.0 × 10^6^ CFU/mL of the test organism was added to appropriately labelled wells and incubated at 37°C. After 24 h after incubation, aliquots (10 *µ*L) were taken from each well and inoculated into freshly prepared 200 *µ*L nutrient broth and incubated at 37°C for 24 h. Twenty microliters (20 *µ*L) of 3-(4,5-dimethylthiazol-2-yl)-2,5-diphenyltetrazolium bromide (MTT) was added after incubation and growth was observed as purple coloration, while clear/yellow coloration indicated no growth. The MBC or MFC was determined as the least concentration of the extract that exhibited no bacterial/fungal growth upon the addition of 20 *µ*L of MTT (1.25 mg/mL). The procedure was carried out in independent triplicates to validate the results.

### 2.10. Time-Kill Kinetics Assay

Time-kill kinetics of methanol extracts of* T. gibbosa, T. elegans, S. commune*, and* V. volvacea* was carried out following the procedure described by Tsuji et al. [48]. Concentrations equal to MIC, twice the MIC, and four times the MIC of the extracts were prepared. An inoculum size of 1.0 × 10^6^ CFU/mL was added and incubated at 37°C. Aliquots of 1.0 mL of the medium were taken at time intervals of 0, 1, 2, 3, 4, 5, 6, 12, and 24 h, for bacteria, and 0, 6, 12, 30, 36, 48, 54, and 72 h for fungi and inoculated aseptically into 20 mL nutrient agar and incubated at 37°C for 24 h. A control test was performed for the organisms without the extracts or reference antibiotic. The colony forming unit (CFU) of the organisms was determined. The procedure was performed in triplicate (three independent experiments) and a graph of the log CFU/mL was plotted against time.

### 2.11. Statistical Analysis

Graph Pad Prism Version 7.0 for Windows (Graph Pad Software Inc., San Diego, CA, USA) was used to analyze data obtained from study by using one-way ANOVA followed by Dunnett's post hoc test.

## 3. Results

### 3.1. Mycochemical Analysis

The methanol extracts of* T. gibbosa*,* T. elegans, S. commune*, and* V. volvacea* were found to contain secondary metabolites such as tannins, flavonoids, triterpenoids, glycosides, and alkaloids. However, coumarins were absent in all four mushroom extracts, and anthraquinones were present in* T. gibbosa, T. elegans*, and* S. commune* but absent in* V. volvacea*, while saponins were absent in* T. gibbosa*,* T. elegans*, and* S. commune* but present in* V. volvacea* (Table 1).

### 3.2. TLC of Methanol Extracts

**Figure 1:**
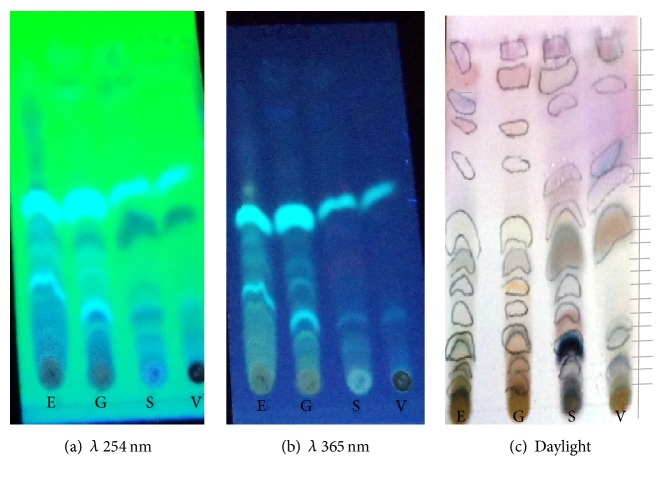
TLC of methanol extracts of mushrooms developed in 100% chloroform. E:* T*.* elegans*; T:* T*.* gibbosa*; V:* V*.* volvacea*; S:* S*.* commune*. (a) Observed under *λ* 254 nm, (b) observed under *λ* 365 nm, and (c) daylight after spraying with anisaldehyde reagent.

The *R*_*f*_ values of the identified bands that eluted from* T*.* gibbosa*,* T*.* elegans*,* S*.* commune*, and* V*.* volvacea* spots on the TLC plates were calculated (Figure 1).* T*.* gibbosa*,* T. elegans*, and* S. commune* extract each resulted in ten (10) bands that eluted from the spot, while seven (7) bands eluted from* V*.* volvacea* extract. However, all four extracts had some bands that were similar as they had the same characteristic fluorescence at both 254 nm and 365 nm (Figure 1).

### 3.3. Antimicrobial Activity of Extracts

#### 3.3.1. Agar Well Diffusion

*T. gibbosa *extract at the highest concentration of 30 mg/mL showed mean zone of growth inhibition of 14.00 ± 1.33 to 20.50 ± 0.55 mm against test Gram-positive bacteria, 20.67 ± 0.82 to 21.12 ± 1.23 mm against Gram-negative bacteria, and 19.50 ± 0.55 mm against* C. albicans* (Table 2).* T. elegans* extract at the highest concentration of 30 mg/mL showed mean zone of growth inhibition of 22.00 ± 1.10 to 14.33 ± 0.82 mm, 23.50 ± 0.55 to 12.67 ± 1.30 mm, and 18.00 ± 0.75 mm against test Gram-positive bacteria, Gram-negative bacteria, and* C. albicans*, respectively (Table 2).* S. commune* extract at the highest concentration of 30 mg/mL showed mean zone of growth inhibition of 21.83 ± 1.17 to 20.50 ± 0.55 mm against test Gram-positive bacteria, 21.60 ± 0.98 to 16.67 ± 0.52 mm against test Gram-negative bacteria, and 21.67 ± 0.52 mm against* C. albicans* (Table 3).* V. volvacea* extract at the highest concentration of 30 mg/mL showed mean zone of growth inhibition of 19.50 ± 0.55 to 16.50 ± 0.55 mm against test Gram-positive bacteria and 22.50 ± 0.55 to 21.17 ± 1.00 mm against test Gram-negative bacteria but showed no activity against* C. albicans* (Table 3). However, all four mushroom extracts showed no inhibition against* A. niger*,* A. flavus*, and* A. tamarii* (Tables 2 and 3).

### 3.4. MIC, MBC, and MFC of Extracts

*T*.* gibbosa* and* T elegans* extracts had antimicrobial activity against test organisms with minimum inhibitory concentration (MIC) ranging from 4 to 20 mg/mL and 6 to 30 mg/mL, respectively (Table 4), while* S. commune* and* V. volvacea* extracts had MIC range of 6 to 20 mg/mL each (Table 5). The MBC of extract of* T. gibbosa* against test Gram-negative and Gram-positive bacteria were between the ranges of 20 to 50 mg/mL, while the MBC of extracts of* T. elegans, V volvacea*, and* S. commune* were between the ranges of 30 to 50 mg/mL each. MFC was only available for the extracts of* T. elegans* and* S. commune* with MFCs of 30 and 50 mg/mL, respectively (Tables 4 and 5).

### 3.5. MBC/MIC and MFC/MIC Ratios of Extracts

MBC/MIC ratios of extract of* T. elegans* against test Gram-negative and Gram-positive bacteria were between the ranges of 5 to 8, while the MBC/MIC ratios of extracts of* T. elegans, V. volvacea*, and* S. commune* were between the ranges of 5 to 6 each. The MFC/MIC ratio was 5 each for extracts of* T. gibbosa* and* S. commune* against* C. albicans*, whereas no MFC/MIC ratio was recorded for the methanol extracts of* T. elegans* and* V. volvacea* (Table 6).

### 3.6. Time-Kill Kinetics of Extracts

**Figure 2:**
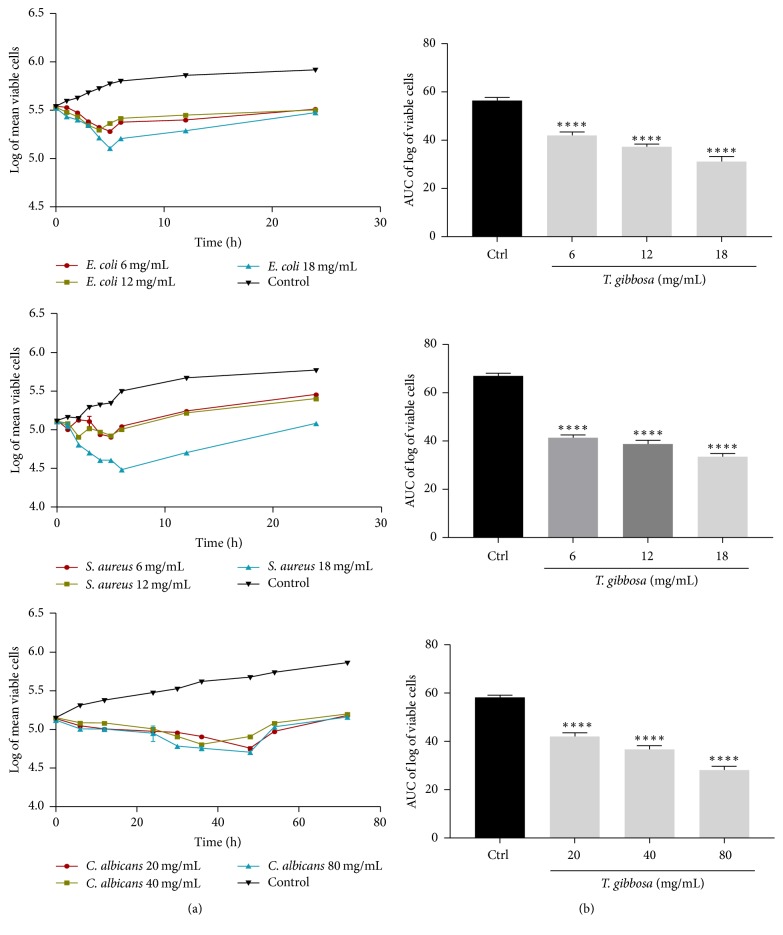
Time-kill kinetics of* T. gibbosa* methanol extract against* E*.* coli*,* S*.* aureus,* and* C*.* albicans*. (a) Time-kill kinetics curve and (b) AUC of time-kill kinetics. Ctrl: control; *n* = 3; values are mean ± SEM. ^*∗∗∗∗*^*p* < 0.0001 compared to the control (one-way ANOVA followed by Dunnett's post hoc test).

**Figure 3:**
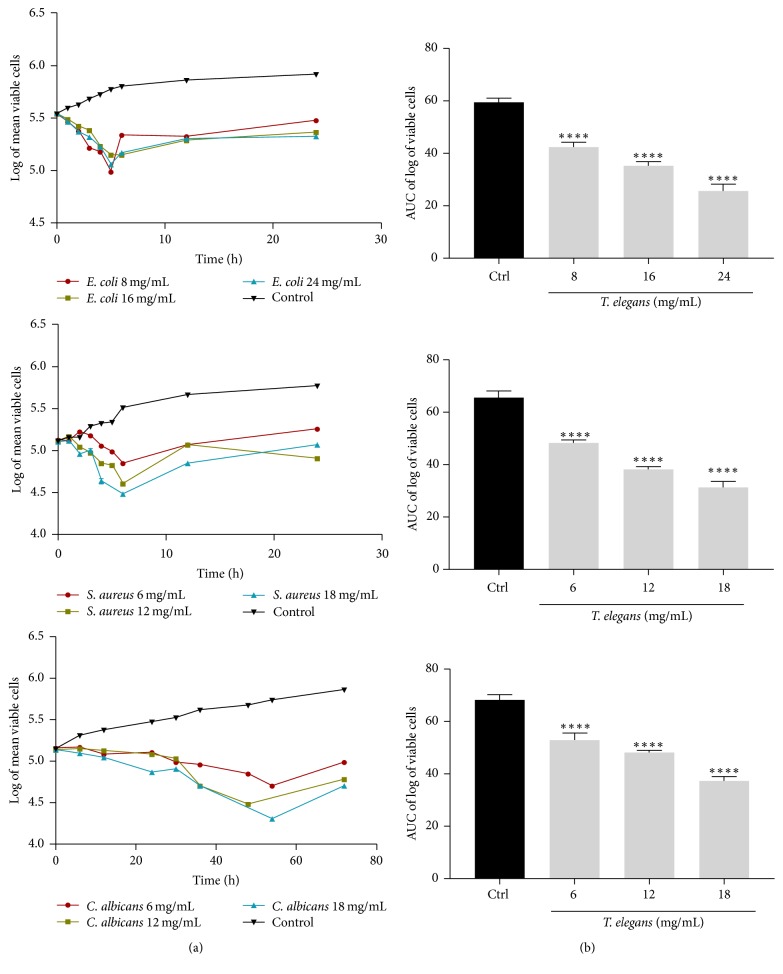
Time-kill kinetics of* T. elegans* methanol extract against* E*.* coli*,* S*.* aureus*, and* C*.* albicans*. (a) Time-kill kinetics curve and (b) AUC of time-kill kinetics. Ctrl: control; *n* = 3; values are mean ± SEM. ^*∗∗∗∗*^*p* < 0.0001 compared to the control (one-way ANOVA followed by Dunnett's post hoc test).

The time-kill kinetics profile of extract of* T*.* gibbosa* against the test organisms* E*.* coli*,* S*.* aureus*, and* C*.* albicans* at test concentrations showed reduction in number of viable cells over the first 5, 6, and 48 hours, respectively, followed by a gradual rise up to the 24th h for* E*.* coli* and* S*.* aureus* and 72nd h for* C*.* albicans* when compared to the control (organisms without antimicrobial agent) (Figure 2). The time-kill kinetics profile of extract of* T*.* elegans* against the test organisms;* E*.* coli*,* S*.* aureus*, and* C*.* albicans* at test concentrations studied showed reduction in number of viable cells over the first 5, 6, and 52 hours, respectively, followed by a gradual rise up to the 24th h for* E*.* coli* and* S*.* aureus* and 72nd h for* C*.* albicans* when compared to the control (Figure 3).

**Figure 4:**
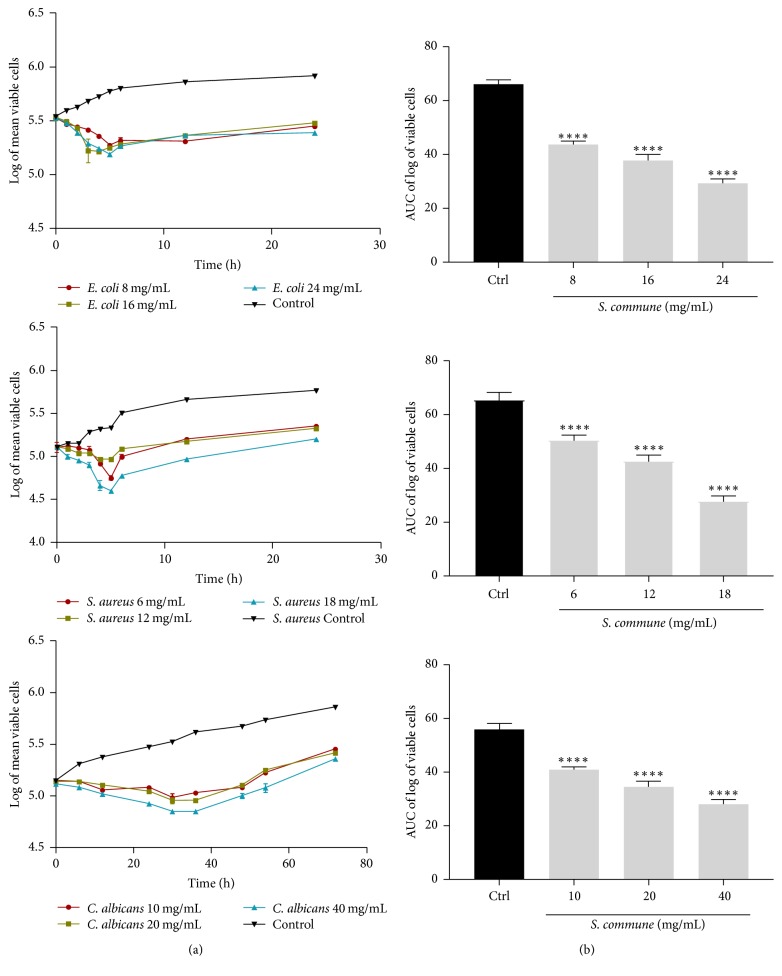
Time-kill kinetics of* S*.* commune* methanol extract against* E*.* coli*,* S*.* aureus*, and* C*.* albicans*. (a) Time-kill kinetics curve and (b) AUC of time-kill kinetics. Ctrl: control; *n* = 3; values are mean ± SEM. ^*∗∗∗∗*^*p* < 0.0001 compared to the control (one-way ANOVA followed by Dunnett's post hoc test).

The time-kill kinetics profile of methanol extract of* S. commune* against the test organisms* E*.* coli*,* S*.* aureus*, and* C*.* albicans* at test concentrations showed reduction in number of viable cells over the first 6, 4, and 36 hours, respectively, followed by a gradual rise up to the 24th h for* E*.* coli* and* S*.* aureus* and 72nd h for* C*.* albicans* when compared to the control (Figure 4).

**Figure 5:**
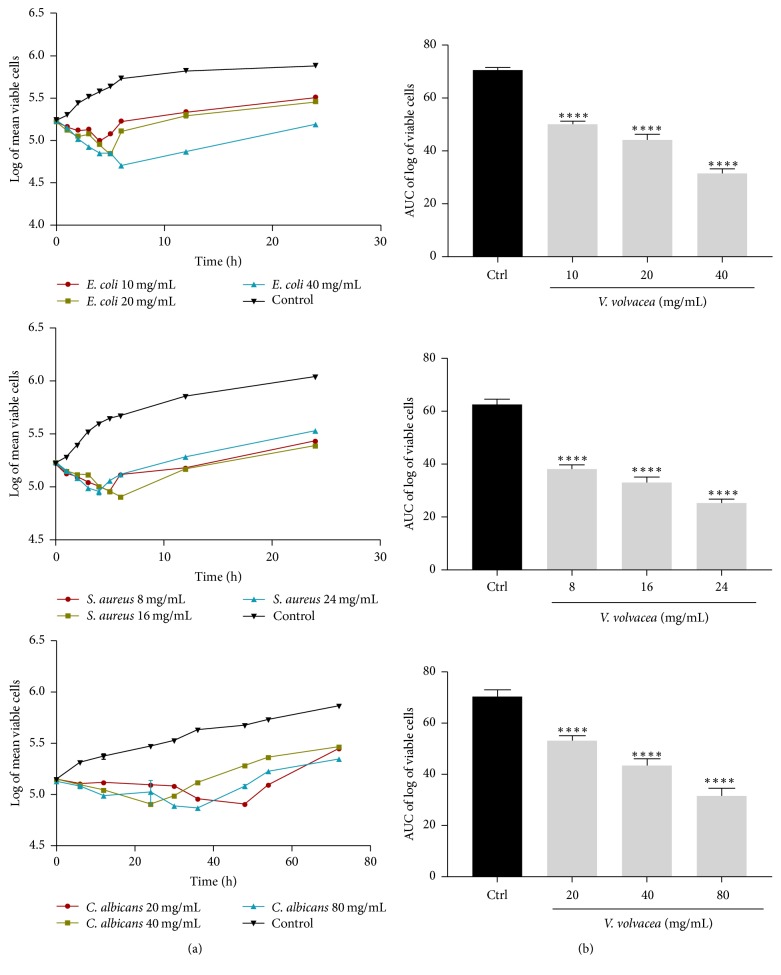
Time-kill kinetics of* V. volvacea* methanol extract against* E*.* coli*,* S*.* aureus*, and* C*.* albicans*. (a) Time-kill kinetics curve and (b) AUC of time-kill kinetics. Ctrl: control; *n* = 3; values are mean ± SEM. ^*∗∗∗∗*^*p* < 0.0001 compared to the control (one-way ANOVA followed by Dunnett's post hoc test).

The time-kill kinetics profile of extract of* V. volvacea* against the test organisms;* E*.* coli*,* S*.* aureus*, and* C*.* albicans* at test concentrations showed reduction in number of viable cells over the first 6, 8, and 42 hours, respectively, followed by a gradual rise up to the 24th h for* E*.* coli* and* S*.* aureus* and 72nd h for* C*.* albicans* when compared to the control (Figure 5). The course of antimicrobial action was however observed to be bacteriostatic and concentration dependent for extracts of all four mushrooms (*T. elegans, T. gibbosa, S. commune*, and* V. volvacea*) studied. The area under the curve (AUC) for* T. gibbosa*,* T. elegans*,* S. commune*, and* V. volvacea* against* E. coli*,* S. aureus*, and* C. albicans* at concentrations studied revealed that the number of cells was significantly (*p* < 0.0001) reduced when compared to the control (Figures 2–5).

## 4. Discussion

Research into the pharmacological activities of mushrooms especially their antimicrobial activity has attracted attention recently. The methanol extracts of* T. gibbosa*,* T. elegans*, and* S. commune* and* V. volvacea* were found to contain secondary metabolites such as tannins, flavonoids, triterpenoids, glycosides, and alkaloids. However, the absence of anthraquinones in* T. gibbosa, T. elegans*, and* S. commune* extracts and the presence of saponins in* V. volvacea* extract (Table 1) may be due to the differences in substrates from which the various samples were collected [49, 50]. The presence of saponins, tannins, terpenoids, flavonoids, and alkaloids in* T. gibbosa* is similar to the report by Mbayo et al. [27] that indicated the presence of tannins, terpenoids, anthocyanins, flavonoids, and alkaloids in* T. gibbosa*. The presence of phenolic compounds in* T. elegans* has been reported by Awala and Oyetayo [14]. The presence of flavonoid in* S. commune* is reported to be responsible for its antimicrobial activity [51].

TLC profile of the extracts, visualized under visible light, indicated the number of bands that eluted from the various extracts. Since silica gel retains the more polar compounds, the nonpolar compounds eluted first and moved further up the TLC plate. Hence, the more polar the compound, the lower the *R*_*f*_ (Retention factor) value and the less polar the compound the larger the *R*_*f*_ value. Thus, it could be said that the mushroom extracts studied have both polar and nonpolar compounds. However, all four extracts (*T*.* gibbosa*,* T. elegans*,* S. commune*, and* V*.* volvacea*) showed bands that were similar as they had the same characteristic fluorescence and *R*_*f*_ value (0.89) (Figure 1). This may be due to the fact they belong to the same class, Basidiomycetes [52], and therefore may possess similar compounds or secondary metabolites.

*T. gibbosa*,* T. elegans*, and* S. commune* and* V. volvacea* have antimicrobial agents or principles present in their extracts though the extent of growth inhibition varied. The antimicrobial activity of methanol extracts of* T. gibbosa*,* T. elegans, S. commune*, and* V. volvacea* may be due to the presence of the secondary metabolites that act individually or in synergism to inhibit the growth of the test organisms [53]. The differences in the diameter zone of inhibition for* T. gibbosa* and* T. elegans* at the highest concentration of 30 g/mL and the next highest concentration of 20 mg/mL (Table 2) may be as a result of the effect of diffusion of the bioactive agents within the medium [54]. Compared with the reference antibiotics,* T. gibbosa* had broad spectrum of activity against the test organisms. This is however in contrast to the findings of Ga and Kaviyarasana [24] who reported that methanol extract of* T. gibbosa* exhibited narrow antibacterial activity against* S. aureus*,* B. subtilis*, and* Micrococcus flavus*, but rather in agreement with the report by Mbayo et al. [27] which revealed that methanol extract of* T. gibbosa* exhibited broad spectrum activity against* Pseudomonas aeruginosa*,* Staphylococcus aureus*,* Streptococcus pneumoniae*, and* Shigella sonnei*.

Methanol extract of* T. elegans* inhibited the growth of all test bacteria (Table 2). Though reports on the antimicrobial activity of* T. elegans* are limited, its inhibitory activity against the test organisms could be attributed to the presence of bioactive compounds and secondary metabolites such as flavonoids, tannins, and triterpenoids [55]. The inhibitory action of* T. elegans* is not surprising as members of the same genus have been reported to possess antimicrobial activity [56].

Microbial growth inhibition was exhibited by extract of* S. commune* against the test bacteria except* B*.* subtilis*. The high zone of growth inhibition exhibited by* S. commune* extract against* C*.* albicans* (Table 3) is in agreement with the results previously reported by Ooi and Liu [57]. The inhibition of growth by extract of* S. commune* against* E*.* coli, S*.* aureus, K*.* pneumonia, P*.* aeruginosa*, and* S*.* pyogenes* is in agreement with the findings of Matsuyama et al. [58].

*V. volvacea *extract inhibited the growth of* S*.* aureus, K*.* pneumoniae, P*.* aeruginosa*, and* S*.* pyogenes* (Table 3) but showed no antimicrobial activity against* E*.* coli* and* C*.* albicans* and this is in agreement with the findings of da Silva et al. [40] that* V. volvacea* exhibits less significant antimicrobial activity, but it exhibited good antioxidant activity. Again, the methanol extracts of all the four mushrooms (*V. volvacea T. elegans, T. gibbosa, and S. commune*) showed no activity against* A. niger*,* A. flavus*, and* A. tamarii* (Tables 2 and 3). This observation supports the findings of Suay et al. [59] and Papadopoulou et al. [60] who reported that polypores and gilled mushrooms are found to exhibit more pronounced antibacterial than antifungal activity.

To overcome the drawbacks of the agar diffusion test including the inability of some extracts to diffuse into agar and to distinguish bactericidal and bacteriostatic effects, the broth dilution method was employed to determine the potency of the extracts. From the MIC results, all four mushroom extracts inhibited the growth of Gram-negative and Gram-positive bacteria (Tables 4 and 5). The above observations support the findings of the broad spectrum antimicrobial activity of some mushrooms. For example,* Pleurotus ostreatus* and* Meripilus giganteus* have been found to exhibit broad spectrum antimicrobial activity [61, 62].

Antimicrobials are usually regarded as bactericidal if the MBC/MIC or MFC/MIC ratio is ≤4 and bacteriostatic if >4 [63]. The ratios obtained for all the test organisms were above 4 which indicated that all four mushroom extracts were bacteriostatic and fungistatic in action against test organisms (Table 6). The bacteriostatic action of these mushroom extracts was also confirmed by the time-kill kinetic studies. Bacteriostatic or fungistatic antimicrobial agents only inhibit the growth or multiplication of pathogenic microorganisms and thus require the host immune system to aid in the elimination of the pathogen [64, 65].

Time-kill kinetic studies indicate that methanol extracts of* T. gibbosa*,* T. elegans*,* S. commune*, and* V. volvacea* exhibited bacteriostatic actions. There are few reports on the time-kill kinetic studies of mushrooms, and several reports of the natural product extracts have been reported [64, 66]. However, the findings in this study are in contrast with the study of Tinrat [67] who determined the time-kill kinetic activity of the mushrooms* Pleurotus sajor-caju, Hypsizygus tessellatus, Lentinus edodes, Flammulina velutipes*, and* Pleurotus eryngii* and found them to exhibit bactericidal activity. There is the need to isolate and characterize the bioactive compounds in the various extracts responsible for the antimicrobial activity.

## 5. Conclusion

Methanol extracts of* T. gibbosa*,* T. elegans*,* S. commune*, and* V. volvacea* exhibited antimicrobial activity but were not active against* A. niger*,* A. flavus*, and* A. tamarii.* Methanol extracts of* T. elegans and T. gibbosa* exhibited static activity against* E*.* coli*,* S*.* aureus*, and* C*.* albicans* and hence there is a need to isolate and purify the agents or molecules from the extracts responsible for the antimicrobial properties which may serve as potential antibiotics.

## Figures and Tables

**Table 1:** Analysis of mycochemical content in extracts of* T. gibbosa*,* T*.* elegans*, *S*.* commune, *and *V*.* volvacea*.

Secondary metabolite	Mushroom samples			
	*T. gibbosa*	*V. volvacea*	*T. elegans*	*S. commune*
Tannins	+	+	+	+
Flavonoids	+	+	+	+
Triterpenoids	+	+	+	+
Alkaloids	+	+	+	+
Coumarins	−	−	−	−
Glycosides	+	+	+	+
Anthraquinones	+	−	+	+
Saponins	−	+	−	−

+: presence of secondary metabolite; −: absence of secondary metabolite.

**Table 2:** Mean zones of growth inhibition of methanol extract of *T. gibbosa, T. elegans, *and reference drugs against test organisms.

Conc. mg/mL	Mean zone of growth inhibition (mm) ± SEM											
	Test organisms											
	PA	EC	SA	BS	SP	KP	EF	ST	CA	AN	AF	AT
*T. gibbosa *extract												

30.0	20.80 ± 0.98	20.67 ± 0.82	17.00 ± 1.03	20.50 ± 0.55	18.83 ± 1.33	21.12 ± 1.33	14.00 ± 1.33	na	19.50 ± 0.55	na	na	na
20.0	21.50 ± 0.84	18.00 ± 0.00	20.00 ± 0.00	20.12 ± 0.41	17.67 ± 0.52	14.50 ± 0.55	12.00 ± 0.00	na	17.33 ± 0.52	na	na	na
10.0	17.50 ± 0.55	na	16.83 ± 0.75	17.00 ± 1.10	14.83 ± 0.41	12.83 ± 0.98	na	na	15.33 ± 0.52	na	na	na
7.5	14.00 ± 1.55	na	15.50 ± 1.05	na	10.00 ± 0.0	na	na	na	na	na	na	na

*T. elegans* extract												

30.0	23.50 ± 0.55	20.33 ± 0.52	22.00 ± 1.10	20.83 ± 0.75	18.00 ± 0.55	22.33 ± 0.52	14.33 ± 0.82	12.67 ± 1.30	18.00 ± 0.75	na	na	na
20.0	20.83 ± 0.41	20.00 ± 0.00	19.33 ± 0.82	20.50 ± 0.55	20.00 ± 0.83	18.00 ± 0.00	na	na	na	na	na	na
10.0	17.67 ± 0.52	15.50 ± 0.55	13.33 ± 0.52	15.50 ± 0.55	16.67 ± 1.30	13.50 ± 1.64	na	na	na	na	na	na
7.5	12.00 ± 0.00	10.67 ± 1.32	12.00 ± 0.00	0.0	15.00 ± 0.00	10.00 ± 0.00	na	na	na	na	na	na

Reference drugs												

Cipro (0.01)	23.17 ± 0.75	20.83 ± 1.00	24.33 ± 1.03	21.00 ± 1.41	25.33 ± 1.03	28.83 ± 1.00	20.25 ± 0.50	21.00 ± 0.0	nd	nd	nd	nd
Keto (0.01)	nd	nd	nd	nd	nd	nd	nd	nd	17.68 ± 0.52	15.33 ± 1.03	17.00 ± 1.00	12.15 ± 0.50

Zones of growth inhibition = diameter of well plus zone of growth inhibition; diameter of well = 8 mm. The mean zone of inhibition was determined from three independent results (*n*) = 3; nd = not determined; na = no activity; SEM = standard error mean; Cipro = ciprofloxacin; Keto = ketoconazole; Conc. = concentration. PA =* Pseudomonas aeruginosa*; EC =* Escherichia coli*; SA = *Staphylococcus aureus;* BS =* Bacillus subtilis*; SP =* Streptococcus pyogenes, *KP =* Klebsiella pneumoniae; *EF = *Enterococcus faecalis*; ST = *Salmonella typhi*; CA =* Candida albicans; *AN = *A. niger*; AF* = A. flavus*; AT= *A. tamarii*.

**Table 3:** Mean zones of growth inhibition of methanol extract of *S. commune*, *V. volvacea, *and reference drugs against test organisms.

Conc. mg/mL	Mean zone of growth inhibition (mm) ± SEM											
	Test organisms											
	PA	EC	SA	BS	SP	KP	EF	ST	CA	AN	AF	AT
*S. commune* extract												

30.0	16.67 ± 0.52	21.60 ± 0.98	21.83 ± 1.17	na	20.50 ± 0.55	19.83 ± 1.33	na	na	21.67 ± 0.52	na	na	na
20.0	15.67 ± 0.82	18.17 ± 1.03	20.33 ± 0.52	na	20.00 ± 0.00	20.50 ± 0.55	na	na	20.00 ± 0.00	na	na	na
10.0	9.00 ± 0.63	12.00 ± 1.33	18.50 ± 0.55	na	17.50 ± 0.84	17.83 ± 1.17	na	na	8.50 ± 0.55	na	na	na
7.5	10.83 ± 0.75	na	16.17 ± 0.41	na	16.33 ± 0.52	16.67 ± 1.03	na	na	na	na	na	na

*V. volvacea *extract												

30.0	21.17 ± 1.00	na	19.50 ± 0.55	16.50 ± 0.55	18.83 ± 1.33	22.50 ± 0.55	na	na	na	na	na	na
20.0	18.67 ± 1.03	na	18.33 ± 0.82	na	17.50 ± 0.55	20.00 ± 0.00	na	na	na	na	na	na
10.0	14.17 ± 1.33	na	17.00 ± 0.00	na	15.00 ± 0.00	17.33 ± 0.52	na	na	na	na	na	na
7.5	12.00 ± 0.00	na	14.33 ± 0.52	na	15.33 ± 0.82	15.33 ± 0.82	na	na	na	na	na	na

Reference drugs												

Cipro (0.01)	23.17 ± 0.75	20.83 ± 1.00	24.33 ± 1.03	21.00 ± 1.41	25.33 ± 1.03	28.83 ± 1.00	20.25 ± 0.50	21.00 ± 0.0	nd	nd	nd	nd
Keto (0.01)	nd	nd	nd	nd	nd	nd	nd	nd	17.68 ± 0.52	15.33 ± 1.03	17.00 ± 1.00	12.15 ± 0.50

Zones of growth inhibition = diameter of well plus zone of growth inhibition; diameter of well = 8 mm. The mean zone of inhibition was determined from three independent results (*n*) = 3; nd = not determined; na = no activity; SEM = standard error mean; Cipro = ciprofloxacin; Keto = ketoconazole; Conc. = concentration. PA = *Pseudomonas aeruginosa*; EC = *Escherichia coli*; SA = *Staphylococcus aureus*; BS = *Bacillus subtilis*; SP = *Streptococcus pyogenes*, KP =* Klebsiella pneumoniae; *EF = *Enterococcus faecalis*; ST = *Salmonella typhi*; CA = *Candida albicans*; AN = *A. niger*; AF =* A. flavus*; AT= *A. tamari*.

**Table 4:** MIC and MBC/MFC of crude methanol extracts of *T. gibbosa*,* T. elegans, *and reference drugs against test organisms.

Test organisms	*T. gibbosa*		*T. elegans*		Cipro	Keto
	MIC (mg/mL)	MBC (mg/mL)	MIC (mg/mL)	MBC (mg/mL)	MIC (*µ*g/mL)	MIC (*µ*g/mL)
*E. coli*	6	30	8	50	≤0.16	nd
*P. aeruginosa*	6	50	8	50	≤0.31	nd
*S. pyogenes *	6	30	8	50	≤0.31	nd
*S. typhi*	8	50	20	—	2	nd
*S. aureus*	6	30	6	30	≤0.31	nd
*K. pneumonia*	6	30	6	50	0.63	nd
*B. subtilis *	4	20	6	30	0.16	nd
*E. faecalis*	8	50	30	—	3.5	nd
*C. albicans*	20	—	6	30	nd	≤0.31
*A. niger*	—	—	—	—	nd	0.35
*A. flavus*	—	—	—	—	nd	0.50
*A. tamarii*	—	—	—	—	nd	2.00

MIC: minimum inhibitory concentration; MBC: minimum bactericidal concentration; nd = not determined; — = MIC/MBC not present / > highest test concentration (50 mg/mL); Cipro = ciprofloxacin; Keto = ketoconazole.

**Table 5:** MIC and MBC/MFC of crude methanol extracts of *V. volvacea*,* S. commune,* and reference drugs (ciprofloxacin and ketoconazole) against test organisms.

Test organisms	*V. volvacea*		*S. commune*		Cipro	Keto
	MIC (mg/mL)	MBC (mg/mL)	MIC (mg/mL)	MBC (mg/mL)	MIC (*µ*g/mL)	MIC (*µ*g/mL)
*P. aeruginosa*	10	50	8	50	≤0.31	nd
*S. pyogenes*	8	50	8	50	≤0.31	nd
*S. typhi*	—	—	—	—	2.00	nd
*S. aureus*	8	50	6	30	≤0.31	nd
*K. pneumonia*	6	30	6	30	0.63	nd
*B. subtilis*	—	—	—	—	nd	nd
*E. faecalis*	—	—	—	—	3.50	nd
*C. albicans*	20	—	10	50	0.16	≤0.31
*A. niger*	—	—	—	—	nd	0.35
*A. flavus*	—	—	—	—	nd	0.50
*A. tamarii*	—	—	—	—	nd	2.00

MIC: minimum inhibitory concentration; MBC: minimum bactericidal concentration; nd = not determined; — = MIC/MBC not present / > highest test concentration (50 mg/mL); Cipro = ciprofloxacin; Keto = ketoconazole.

**Table 6:** MBC/MIC and MFC/MIC ratios of methanol extracts of *T. gibbosa*,* T*.* elegans*, *S*.* commune, *and *V*.* volvacea* against test organisms.

Test organisms	MBC/MIC and MFC/MIC ratio			
	*T. gibbosa*	*T*.* elegans*	*S*.* commune*	*V*.* volvacea*
*E. coli*	5	6	5	na
*P. aeruginosa*	5	6	6	5
*S. pyogenes*	5	6	6	6
*S. typhi*	6	na	na	na
*S. aureus*	5	5	5	6
*K. pneumonia*	5	8	5	5
*B. subtilis*	5	5	na	na
*E. faecalis*	6	na	na	na
*C. albicans*	na	5	5	na
*A. niger*	na	na	na	na
*A. flavus*	na	na	na	na
*A. tamarii*	na	na	na	na

na = MBC/MIC or MFC/MIC ratio not available.
